# The Common Accidents of Every-Day Life

**Published:** 1888-01-28

**Authors:** Robert A. P. Forrester


### THE COMMON ACCIDENTS OF EVERY-DAY LIFE.

By ROBERT A. P. FORRESTER, B.A.Dub., M.B. and C.M.Edin.

**Fractures and Dislocations.**

THESE accidents, as all are aware, are very common, and it is of importance that people should have some idea of the temporary management of such cases when suddenly brought face to face with them. A policeman in Liverpool, some few months ago, was able to render such valuable assistance to a gentleman who had broken his leg in the streets, by applying a temporary splint in so skilful a manner that the surgeons said he had probably prevented the occurrence of a compound fracture. This latter might have taken place had the limb been roughly handled by an inexperienced person. The policeman had attended a course of ambulance lectures, and had thus turned his knowledge to good account.

It is within the power of a large majority of the people to obtain *some* knowledge of what to do in the "common accidents of every-day life," and I am not asserting too much when I say that many a non-professional person may be the means of saving a fellow-creature's life, or at least of rendering such help as may to a great extent alleviate suffering. I shall give one instance in which the exercise of a little common-sense combined with a little knowledge might have been successful, but unfortunately neither the common-sense nor the knowledge was there. A young lady, who was the subject of varicose veins, on getting out of bed one morning, hit one of her legs against the framework, and wounded the skin so severely over the course of one of the swollen veins that it burst and bled profusely. She fell on the floor, and the blood continued to flow very freely. Her friends were aroused by the noise of her fall, and on seeing her condition could think of nothing better to do than to cover her up and send for the doctor. When he arrived the lady was dead. The blood had continued to flow without check, and in a very short time a fatal result took place. It certainly was a very alarming accident, and perhaps the fright thereby occasioned was excusable. What, however, *might* have been done? If anyone had had the common-sense to put the finger or thumb over the spot whence the blood was coming, and perhaps, in addition, had raised the leg a little and "*held on*" until the doctor's arrival, the patient's life would have been saved. If a water pipe bursts, the opening is generally tied up roughly until the plumber arrives. Why cannot something of the kind be done when a similar accident happens to a human being? I shall allude to this case again when we speak of "Hæmorrhage, and its management."

But to return to fractures and dislocations! What is a fracture? and what is a dislocation? People are generally frightened at the long names given to diseases and injuries, and wonder why medical men do not make use of their own language in speaking and writing of them. There are various reasons for using the Greek and Latin languages in medical science one being that we can express by one Greek or Latin word what would require more in our own tongue; another, that medical men of all nations have agreed to use Greek and Latin to exchange ideas, much in the same way as Governments use French for correspondence: *e.g.*, "perihepatitis" means inflammation of the surrounding capsule of the liver—*peri*, round; *hepar*, liver; *itis*, inflammation. An English surgeon understands that, and so does a Japanese. It is an English medical term, as it is a Japanese medical term. *Fracture* has been called the *solution of continuity in any bone;* and *dislocation*, the separation of the articular surfaces of two bones from each other. I cannot say which you will prefer—the above terms, or their definition. It is necessary, however, to explain by words more generally known the meaning of others not so well understood. It will be as well, before treating of any particular fracture or dislocation, to say something about these accidents and to take them together. First, as to the general symptoms: in all fractures we have the following—shortening of the limb, and distortion, with pain and swelling; unnatural mobility or movement, and a peculiar crackling sensation, or "crepitus," as it is called, when the surgeon rubs the surfaces of the broken bone together. This last is a most valuable sign, but it is not always possible to get it. Compare now the symptoms of dislocation: there we have, as in fracture, distortion, also swelling and pain; but we cannot possibly get crepitus, and the limb, instead of being unnaturally mobile, is quite the contrary.

(*To be continued.*)

